# TIMP-1 is an activator of MHC-I expression in myeloid dendritic cells with implications for tumor immunogenicity

**DOI:** 10.1038/s41435-024-00274-7

**Published:** 2024-05-22

**Authors:** Miriam Langguth, Eleftheria Maranou, Saara A. Koskela, Oskar Elenius, Roosa E. Kallionpää, Eva-Maria Birkman, Otto I. Pulkkinen, Maria Sundvall, Marko Salmi, Carlos R. Figueiredo

**Affiliations:** 1 Medical Immune Oncology Research Group (MIORG), Institute of Biomedicine, Faculty of Medicine, University of Turku, Turku, Finland; 2 Auria Biobank, University of Turku and Turku University Hospital, Turku, Finland; 3 Department of Pathology, Laboratory Division, Turku University Hospital and University of Turku, Kiinamyllynkatu 10, 20520 Turku, Finland; 4 Cancer Research Unit, Institute of Biomedicine, and FICAN West Cancer Center Laboratory, University of Turku, and Turku University Hospital, Kiinamyllynkatu 10, 20520 Turku, Finland; 5 Department of Oncology, Turku University Hospital, Turku, Finland; 6 InFLAMES Research Flagship Center, University of Turku, Turku, Finland; 7 Institute of Biomedicine, University of Turku, Turku, Finland; 8 MediCity Research Laboratory, University of Turku, Turku, Finland; 9 Turku Bioscience Centre, University of Turku and Åbo Akademi University, Tykistökatu 6, 20520 Turku, Finland

**Keywords:** Antigen-presenting cells, MHC

## Abstract

Immune checkpoint therapies (ICT) for advanced solid tumors mark a new milestone in cancer therapy. Yet their efficacy is often limited by poor immunogenicity, attributed to inadequate priming and generation of antitumor T cells by dendritic cells (DCs). Identifying biomarkers to enhance DC functions in such tumors is thus crucial. Tissue Inhibitor of Metalloproteinases-1 (TIMP-1), recognized for its influence on immune cells, has an underexplored relationship with DCs. Our research reveals a correlation between high TIMP1 levels in metastatic melanoma and increased CD8 + T cell infiltration and survival. Network studies indicate a functional connection with *HLA* genes. Spatial transcriptomic analysis of a national melanoma cohort revealed that *TIMP1* expression in immune compartments associates with an HLA-A/MHC-I peptide loading signature in lymph nodes. Primary human and bone-marrow-derived DCs secrete TIMP-1, which notably increases MHC-I expression in classical type 1 dendritic cells (cDC1), especially under melanoma antigen exposure. TIMP-1 affects the immunoproteasome/TAP complex, as seen by upregulated PSMB8 and TAP-1 levels of myeloid DCs. This study uncovers the role of TIMP-1 in DC-mediated immunogenicity with insights into CD8 + T cell activation, providing a foundation for mechanistic exploration and highlighting its potential as a new target for combinatorial immunotherapy to enhance ICT effectiveness.

## Introduction

Immune checkpoint therapy (ICT) has transformed advanced solid tumor treatment. In melanoma, combined anti-PD-1 (pembrolizumab, nivolumab) and anti-CTLA4 (ipilimumab) therapies result in a five-year survival rate of 52% [1, 2]. Yet, many metastatic melanoma patients either resist or relapse post-ICT, with only the ones with BRAF mutation in tumors qualifying for targeted therapies as a standard of care [3]. Approximately 53% of these patients show ICT resistance due to T cell exclusion mechanisms [4]. Such “cold” tumors, characterized by a nonimmunogenic tumor microenvironment (TME) and minimal immune infiltration, are invariably resistant to ICT [5]. Recently, we discussed how cancer cells foster these cold tumors, via T cell desertification, where they regulate suppressive elements inhibiting dendritic cells (DCs) from effectively presenting tumor neoantigens to T cells, causing reduced anti-tumor T cell expansion [6]. This compromised DC functionality can culminate in immune-desert tumors, marked by a dearth of anti-tumor immunity and unresponsiveness to ICT [7].

Research efforts have been placed on identifying new pathways and biomarkers that enhance the immunogenic activities of DCs in the TME [8, 9]. To this end, combinatorial methods have been developed, leveraging the discovery of these biomarkers. These approaches often involve strategies like drug infusions or gene therapies to elevate their presence in the TME [9]. In this context, the Tissue Inhibitor of Metalloproteinases-1 (TIMP-1) has recently emerged as a potential immune functional biomarker [10]. While traditionally recognized for its primary role in inhibiting metalloproteinases [11], recent findings highlight TIMP-1 as a promising cytokine interacting with multiple cell-surface receptors and co-receptors, including CD63, LRP1, and CD74 [12–14]. Notably, all these receptors have been previously described to play critical roles in the immunogenic functions of myeloid DCs [15–17]. However, the association of TIMP-1 with immunogenic responses in cancers, particularly in metastatic melanoma, encompassing features of DC activation such as antigen presentation, remains unexplored.

In this study, by integrating spatial-immune monitoring from clinical datasets and reverse translation [18], we delved into a multidisciplinary study of TIMP-1’s role in the immunogenic context of metastatic melanoma, a cornerstone model for ICT research [19, 20]. In the GDC-TCGA-SKCM melanoma dataset, *TIMP1* expression correlated with better survival, increased CD8 + T-cell infiltration, and expression of pivotal *HLA* genes. Using NanoString GeoMX, we found *TIMP1* expression in the immune compartment of the TME, which corresponded to higher immunogenicity in lymph nodes (LN) due to its association with MHC-I peptide loading, indicating TIMP-1’s possible impact on melanoma immunogenicity through DC priming. We further demonstrated that both T cells and DCs secrete TIMP-1 and that its soluble form increased the expression of MHC-I in myeloid DCs, exposed to tumor antigens, highlighting its potential to enhance antigen presentation in DCs, particularly in classical type 1 dendritic cells (cDC1). Initial mechanistic investigations reveal that TIMP-1 exerts modulatory effects on PSMB8 and TAP-1, constituents of the immunoproteasome/TAP complex. These findings are consistent with the functional associations predicted by cross-tissue spatial analysis within our national melanoma cohort and preliminary cross-presentation and T cell activation studies. Our study seeks to position TIMP-1 as a new immune biomarker for the future development of optimal combinatorial approaches that unleash tumor immunogenicity and ICT benefits.

## Materials and methods

### Human subjects

Tissue Micro Arrays (TMA) were created from archived formalin-fixed paraffin-embedded (FFPE) melanoma samples, which were selected based on inclusion criteria of untreated patients with a histologically confirmed diagnosis of metastasized cutaneous melanoma at the Turku University Hospital from 2011 to 2019. Hematoxylin & Eosin (H&E) staining was employed to identify and select tumor regions with immune cell infiltrates, guiding the precise selection of intersecting areas of tumor and immune cells for subsequent TMA construction and detailed spatial analysis by NanoString GeoMX. Selection criteria were informed by comprehensive pathology reports and electronic health records, focusing on the most diagnostically relevant areas of the tissue specimens. Following this, a 1 mm diameter core from each designated tumor block was precisely extracted and arrayed using an automated tissue arrayer (TMA Grand Master, 3DHISTECH Ltd., Budapest, Hungary), ensuring the integrity and uniformity of the sample cores within the TMA. The samples were provided for NanoString spatial Next Generation Sequencing (NGS) analysis as 3,5 μm TMA sections. Patients whose tumors were not detected in the nanostring analysis were further excluded from the study.

### GDC-TCGA analysis

RNA-seq and clinical data for metastatic melanoma patients were sourced from the GDC PANCAN datasets, including the metastatic cutaneous melanoma SKCM cohort (*n* = 456) (Supplementary Table 1), via the UCSC Xena browser (http://xena.ucsc.edu) as previously described [21, 22]. Kaplan-Meier survival analysis examined high vs. low mRNA expressions of *TIMP1* and *CD8A*, noting significant disparities at *p* < 0.05. The survival cutoff was established at the median of normalized expression. Gene correlational studies were assessed using Spearman’s correlation test [23]. Normalized [log2(fpkm-uq+1)] gene expression, overall survival (OS), and OS time were derived from the GDC-TCGA-PANCAN dataset. A heatmap qualitatively displayed gene expression, with *TIMP1* expression supervising patient distribution. *HLA* genes were obtained from previously described NanoString datasets [21] and upgraded via STRING network analysis (https://string-db.org/) as previously described [24], which were further included in the heatmap.

### Spatial NGS analysis from metastatic melanoma tumors

Whole transcriptome analysis of LNs and matched skin biopsies from metastatic melanoma patients was conducted using the NanoString Spatial Geo MX Whole Transcriptome Atlas RNA probe kit (NanoString Technologies, Seattle, WA, USA) following manufacturer’s guidelines with some modifications [23]. To ensure the inclusion of samples that distinctly presented both immune and tumor segments within the same biopsy, we included *n* = 37 LN patient samples, *n* = 35 skin patient samples, and *n* = 11 patients with both skin and LN metastatic samples. All samples included in this study that were originated from skin tumors or tumor-draining LN and were confirmed to have tumors. To evaluate the spatial distribution of melanoma and immune infiltrating cells, we utilized the GeoMx Melanoma Morphology Kit HsR and HsP (for both RNA and Protein) (NanoString Technologies, Seattle, WA, USA). The kit enables the identification of areas predominantly occupied by tumor cells (PMEL17 + , green), and immune cells (CD45 + , red). In our particular study, we obtained and analyzed only transcriptomic areas from immune cells (CD45 + ), prioritizing immune cells near the interface with tumor cells. In brief, the following workflow was used: deparaffinization of 3.5 µm-thick FFPE sections, antigen retrieval, and overnight RNA probe hybridization. Post-hybridization, the slides were stained using the Human Melanoma TME morphology kit antibodies (NanoString Technologies) to identify Regions of Interest (ROIs). For some patients, more than one ROI was taken for analysis. These were visualized on the NanoString Geo MX instrument (Karolinska Institute), emphasizing only immune ROIs (iROIs). RNA-specific probes from iROIs were prepared for next-generation sequencing.

### *TIMP1* cross-tissue spatial and reactome analysis

We analyzed transcriptomes from iROIs of eleven metastatic melanoma tumors and their matched LN, and the transcriptomes of 51 LNs from 30 metastatic melanoma patients. Because some TMA cores varied in size, we acquired and analyzed multiple TMA cores for certain patients to ensure consistency in the transcriptomic areas examined across the entire cohort. Correlating *TIMP1* differential expression in skin iROIs with LN transcriptomes using Spearman’s test yielded a gene signature, cT1S, significant at p < 0.05. cT1S underwent pathway enrichment analysis using Gene Ontology Consortium, Reactome version 85 (Released 2023-05-25), and PANTHER and Overrepresentation Test with a BINOMIAL test [25]. We prioritized the top 20 expected pathways with significant enrichment scores to minimize chance findings. These pathways were categorized into seven areas: Cellular Transport, Intracellular Responses, and others. Their average fold enrichment is plotted in bar plots.

### Cell lines, culture, and conditioned media

B16F10 murine melanoma cell line was recently purchased from the American Type Culture Collection (ATCC, CRL-6475) with recent appropriate profiling certification and cultured in DMEM (Gibco, Billings, MT, United States) supplemented with 10% Newborn Calf Serum (NBCS) (Gibco), 4 mM L-Glutamine (Gibco), and 1% penicillin-streptomycin (P/S) (Gibco). Cell lines were periodically tested for mycoplasma contamination. Conditioned media from B16F10 (EV-TCM), was produced by culturing the cells in DMEM, supplemented with 2% NBCS. Once the cells reached confluency, EV-TCM was collected, centrifuged at 2000 x g for 4 minutes, and filtered using a 0.2 µm disc filter.

### Mice and ex vivo culture of BMDCs

C57BL/6 J mice (*Mus musculus*) were purchased from The Jackson Laboratory (Bar Harbor, ME, USA). BMDCs were obtained from 6 to 8-week-old C57BL/6 J male mice, as described earlier [17, 26] with some modifications. In brief, bone marrow was flushed from femora, and red blood cells were lysed with red blood cell lysis buffer (Sigma-Aldrich, St. Louis, USA). The cells were cultured in RPMI-1640 medium (Gibco) supplemented with 10% NBCS (Gibco), 1% P/S, and 150 ng/mL 2-mercaptoethanol, and 200 ng/mL mouse FLT3 ligand (FLT3L) [27] (Sino Biological, Beijing, China), the latter boosting tumor antigen presentation for anti-PD1 responses [28]. Medium was replaced every three days.

### Primary human peripheral blood mononuclear cell culture

Human peripheral blood mononuclear cells (PBMCs) were isolated from buffy coats upon arrival, using a histopaque-1077 (Sigma-Aldrich) gradient, washed with PBS, and red blood cells were similarly depleted. T cells and monocytes were isolated from frozen PBMCs by negative selection using human CD3 microbeads (Miltenyi Biotech, Bergisch Gladbach, Germany) and positive selection using a monocyte isolation kit (Miltenyi Biotech) respectively. Both cell types were cultured in RPMI-1640 medium (Gibco) supplemented with 10% NBCS (Gibco), 1% P/S, and 150 ng/mL 2-mercaptoethanol. Monocytes were differentiated into DCs using 20 ng/mL human IL-4 (Miltenyi Biotec), and 50 ng/mL recombinant human GM-CSF (Miltenyi Biotec), with the medium being replaced every 3 days.

### Primary cultures and stimulation conditions

For functional studies, BMDCs were incubated with 100 ng/mL of recombinant mouse TIMP-1 (R&D Systems, Minneapolis, MN, USA, #980-MT) alone, or in combination with 50% EV-TCM from B16F10 cells. The recombinant TIMP-1 used is widely characterized for its suitability for functional assays and very low endotoxin levels ( < 0.01 EU/μg), as demonstrated in previous publications [29–31]. However, an additional endotoxin-negative control was performed using a heterologous recombinant human TIMP-1 (R&D Systems, Minneapolis, MN, USA, #970-TM). After 24 h incubation with the recombinant proteins, the BMDCs were harvested, washed with PBS, centrifuged at 1000 rpm for 5 minutes, and stained for immunophenotyping studies using flow cytometry, or lysed using lysis buffer [17] (Cell Signaling Technology, Danvers, MA, USA) to be used for cell signaling assays using Western blot. For evaluating the levels of secreted TIMP-1 in the primary cultures, BMDCs were left untreated or stimulated with 100 ng/mL of LPS from *E.coli* (Sigma-Aldrich) for 24 h as described previously [32]. The obtained BMDC-conditioned media was collected as described above and used for Western Blot. Primary human T cells were stimulated with Dynabeads Human T-Activator CD3/CD28 (Thermo Fisher Scientific, Waltham, MA, USA, 11161D) on day 0 for 3 days. Primary human DCs were stimulated on day 7 using 10 ng/mL LPS from *E.coli* (Sigma Aldrich) and 1000 IU/mL recombinant human IFN-gamma (IFN-γ) (Sigma Aldrich) as previously described [33] for 24 h. Conditioned media was collected as described above to be used for evaluation of TIPM-1 using Western blot.

### Protein lysates and western blot

For quantification of soluble TIMP-1 levels, media from mouse BMDCs, human DCs, and T cell cultures were concentrated using >3 kDa Amicon ultra-centrifuge 0.5 M filter (Millipore, USA) as previously described [34]. Protein lysates from cells and Western blots were performed as previously described in [17]. Protein concentrations were determined using the Pierce BCA protein assay kit (Thermo Fisher Scientific). Each sample (30 µg of protein) was run on a 4–20% Criterion TGX gel (Bio-Rad, Hercules, CA, USA) post 95°C incubation for 5 minutes. The Trans-Blot Turbo (Bio-Rad) transferred proteins to a nitrocellulose membrane. After blocking with 5% milk in TBS-T, the membrane was incubated overnight with primary rabbit anti-TIMP-1 antibody (Nordic BioSite, ASJ-9241OJ-50), rabbit anti-TAP-1 (Cell Signaling Technology, 49671), rabbit anti-PSMB8 (Cell Signaling Technology, 13635), rabbit anti-calreticulin (Cell Signaling Technology, 12238), or rabbit anti-GAPDH (Cell Signaling Technology, 2118 S) and then with anti-rabbit IgG HPR-linked secondary antibody (Cell Signaling Technology, 7074P2). Protein levels were measured using the enhanced chemiluminescence reagent Luminata Forte (Merck, Darmstadt, Germany) and visualized using ChemiDoc (Bio-Rad). Loading control analysis was performed using a Ponceau staining solution.

### Flow cytometry assays

To measure the levels of MHC-I, mouse cells were stained for flow cytometry, as previously described with some modifications [17]. Briefly, each sample was divided equally and incubated with PE-conjugated anti-mouse MHC-I (Invitrogen, Waltham, MA, USA, 12-5958-82) and PE-conjugated anti-mouse MHC-II (Invitrogen, 12-5321-82) at +4 ^o^C in the dark for 40 min. For the phenotyping of the BMDCs, the cells were incubated with the following antibodies: CD11b-Briliant Violet 421 (Biolegend, San Diego, CA, USA, 101235), CD86-FITC (Biolegend, 105006), Ly-6G/Ly-6C-PE/Cyanine7 (Biolegend, 108416), CD24-APC (Biolegend, 101814), B220-Pacific Blue (Biolegend, 103227), CD11c-Alexa Fluor® 700 (Biolegend, 117320). After two washes with PBS, samples were resuspended in PBS + 2% BSA. The events were acquired using an LSRFortessa Jazz (BD, Franklin Lakes, NJ, USA) flow cytometer. For multi-color characterization of cDC1s, the cells were resuspended in PBS and samples were acquired using a three-laser Cytek Northern Lights Flow cytometer (Cytek Bioscience, San Diego, CA, USA) as previously described [35] using the SpectroFlo version 2.2.0 software and further analyzed with FlowJo (BD Biosciences).

In our methodology, we employed Hierarchical Stochastic Neighbor Embedding (HSNE) analysis using Cytosplore software [36] to robustly cluster and visualize distinct cell populations expressing varying levels of MHC-I. This approach was chosen due to its ability to analyze multidimensional data, encompassing parameters such as FSC-A, FSC-H, SSC-A, SSC-H, and MHC-I from three independent experiments, as previously described [23]. HSNE’s hierarchical data reduction technique preserves both local and global structures, enabling the differentiation of cell populations at multiple granularity levels of MHC-I expression, not often distinguishable bi-dimensional scatter plots. Moreover, Median Fluorescence Intensity (MFI) was alternatively calculated for MHC-I (PE) excluding background fluorescence in the PE channel within the total CD11c+ cells and conventional type 1 DCs (cDC1s), characterized as B220-CD11 + CD24 + , with FlowJo. Relative MFI revealing fold-change was calculated for all samples using: Relative MFI = MFI (sample)/MFI (negative control).

### Dendritic cell/T-cell cross-presentation activation assay

CD8 + T cell proliferation was assessed by co-culture, as described before [37], with modifications. In brief, FLT3L-differentiated BMDCs were treated with 100 ng/mL of TIMP-1 (R&D Systems) for 24 h. Mouse spleens were collected, and passed through a 70 µm mesh filter, and erythrocytes were depleted using red blood cell lysis buffer. Then, the single cells were enriched for CD8 + T cells by negative selection using a CD8a+ T cell isolation kit (Miltenyi Biotech), according to the manufacturer’s instructions. T cells were stained with 1 µM CellTracker™ Green CMFDA Dye (Thermo Fisher Scientific) according to the manufacturer’s instructions and added to the DCs at a ratio of 40,000 DC to 160,000 CD8 + T cells and incubated in the presence or absence of 100 µg/mL ovalbumin protein (Invivogen, San Diego, USA), and 30 U/mL IL-2 (Peprotech, London, United Kingdom) for three days. Following the incubation, T cells were collected by pipetting, stained with ViaDye Red fixable viability dye (Cytek) and anti-mouse CD3 antibody conjugated to PE (Biolegend), and acquired on a Cytek flow cytometer. IL-2 levels were quantified from undiluted cell culture supernatants using Mouse IL-2 ProQuantum Immunoassay Kit (Thermo Fisher Scientific, A42892) [38] following the manufacturer’s guidelines with some modifications. In brief, the IL-2 levels were detected in qPCR using C1000 Touch Thermal Cycler and CFX96 Touch Real-Time PCR Detection System (Bio-Rad) and settings protocol for standard block type. A linear regression model, fitted to the IL-2 standard curve, was used for interpolation of *Cq* values for concentration determination. The standard curve was linearized by inspecting logarithmic fold changes in the concentration values. Measured *Cq* values and standard curve were normalized to a [0,1] range for comparison. Linear regression on the standard curve estimated log-fold changes in concentrations for all experimental conditions. Concentration levels are presented in pg/mL and were normalized to total protein content measured by Pierce BCA Protein Assay Kit (Thermo Fisher Scientific) following the manufacturer’s instructions.

### Statistical analysis

**Fig. 1:**
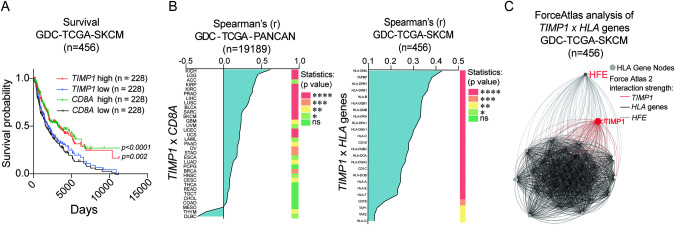
Correlation of *TIMP1* gene expression with improved survival, T-cell infiltration, and antigen presentation in melanoma. **A** Kaplan-Meier plots of overall survival (OS) based on *TIMP1* (red and blue) and *CD8A* (green and black) gene expressions (cutoff determined by the median of gene expression within the cohort), demonstrating significant differences in the OS curves determined by *TIMP1* and *CD8A* differential expression. **B**, left Spearman’s correlation screening of *TIMP1* with *CD8A* gene expression in all cancer cohorts of the normalized GDC-TCGA-PANCAN dataset. The heatmap represents the significance of correlations based on a two-tailed test. **B**, right Quantitative Spearman’s correlation between *TIMP1* and significant *HLA*-related genes (*p* < 0.05); in red, genes controlling peptide loading onto MHC-II and MHC-I molecules (****p < 0.0001, ***0.0001<*p* < 0.001, **0.001<*p* < 0.01 and *0.01<*p* < 0.05 are considered significant). **C** Force Atlas 2 Network study displaying genes as nodes and correlations as edges, with correlation strength indicated by edge distance, revealing *TIMP1*’s stronger relationships with *HLA* genes compared to *HFE*.

**Fig. 2:**
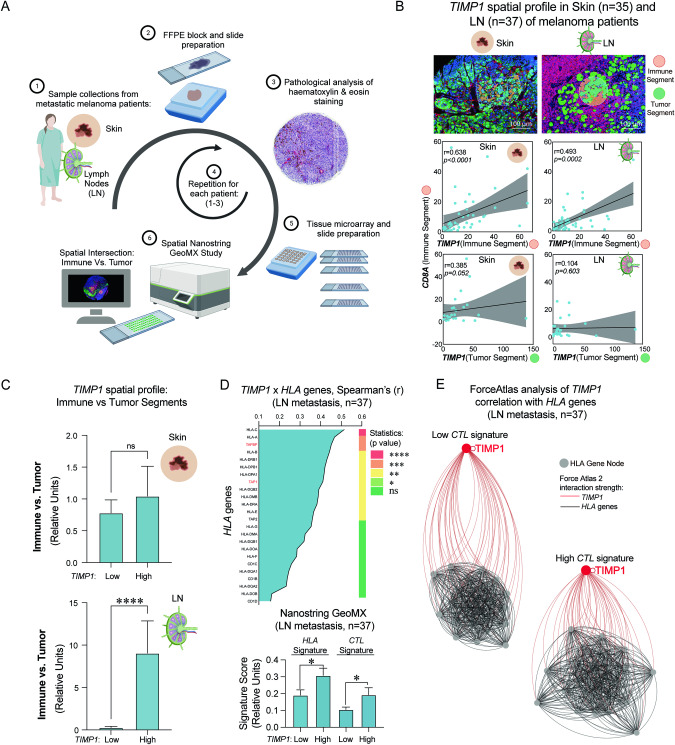
*TIMP1* spatial expression profile in skin melanomas and tumor-draining lymph nodes of metastatic melanoma patients. **A** Schematic representation of biobank study for spatial expression profiling of *TIMP1* in metastatic melanoma. (1) Skin tumors and lymph node metastasis from melanoma patients were collected and their (2) histopathology was analyzed to select areas of immune and tumor intersection (3). (4) The process is repeated for each participant of the study. (5) TMA blocks are built by collecting the selected cores and slides were prepared for further staining for NanoString study (6). **B** Whole transcriptome analysis of iROIs of skin (*n* = 35) and LN (*n* = 37) biopsies of metastatic melanoma patients. Upper panel: representative immunofluorescence images obtained from GeoMx Melanoma Morphology Kit for tumor cells (PMEL17+ green), immune cells (CD45+ red), and nuclei counts (Blue). Lower panels: Spearman’s rank of normalized *TIMP1* counts obtained from tumor (green) and immune (red) segments to *CD8A* counts obtained from the immune segment (red). **C** Spatial ratio profiling of *TIMP1* expression in the immune segment relative to the expression levels in the tumor segment in both skin and LN in patients with high and low expression of *TIMP1*. Two-tailed Maan–Whitney test was performed. **D** Spearman’s correlation analysis displays the relationships between *TIMP1* expression and corresponding HLA genes in LN metastasis from Auria cohort. Genes are sorted from the highest to lowest correlation score. The upper panel presents the highest correlation coefficients associated with *HLA-A*, *-B*, *-C*, *TPBP*, and *HLA-DPA*. The heatmap represents the significance of correlations based on a two-tailed test. ****p < 0.0001, ***0.0001<*p* < 0.001, **0.001<*p* < 0.01 and *0.01<*p* < 0.05 are considered significant. The lower panel demonstrates both the *HLA* and *CTL* gene signature expression according to *TIMP1* levels in the cohort. Two-tailed Maan-Whitney test was performed. **E** Force Atlas 2 analysis of *TIMP1* interaction strength with *HLA* gene nodes within the lymph node dataset. *TIMP1* node and interaction strings are highlighted in red, revealing its stronger proximity with HLA gene nodes in patients displaying high levels of cytolytic signature (CTL) within the immune segments, compared to those with low CTL levels.

In our study, all available samples from the biobank were utilized. The statistical power of our sample size was confirmed using G*Power software as previously described [39]. The power analysis was anchored on an effect size (∣ρ∣) of 0.42, which reflects the highest observed Spearman’s correlation between *TIMP1* and *HLA* genes in the GDC-TCGA dataset. With a set significance level of 5% and aiming for 80% power, our analysis indicated that a minimum of 30 patients would provide adequate statistical power for the study. Consequently, our analysis included 30 skin tumor and 37 lymph node (LN) samples. GraphPad Prism software was used for statistical analysis. The Spearman’s correlation was used to assess the correlation of *TIMP1* with *CD8A* as well as with genes belonging to the *HLA* family. For correlative Ladder Plots from Figs. 1 and 2, correlations were considered significant when *0.01<*p* < 0.05; **0.001<*p* < 0.01, ***0.0001<*p* < 0.001 and ****p < 0.0001. UCSX Xena browser was used to obtain the Overall Survival (OS) data from melanoma patients according to gene differential expression (median cutoff), and Kaplan-Meier survival plot and the log-rank test were performed to evaluate the significance of OS curves using GraphPad Prism Version 9. Statistical analysis of *TIMP1* expression across tissue sections, group signature comparisons, and functional BMDC studies with recombinant TIMP-1 employed the two-tailed unpaired *t* test for normally distributed data, determined by the Shapiro-Wilk test. At least three biological repetitions were performed for all functional studies. For non-normally distributed data, the Mann-Whitney test was utilized. The homogeneity of variances was checked using Welch’s correction where necessary, ensuring accurate comparisons between groups. P values ranges are detailed in the figure captions.

## Results

### *TIMP1* gene expression is associated with improved survival, enhanced T cell infiltration, and antigen presentation in melanoma

Using a reverse translational approach, we initially assessed the prognostication power of *TIMP1* in comparison with *CD8A* in the GDC-TCGA-SKCM melanoma dataset [40], a cornerstone model for ICT research [19, 20]. As shown in Fig. 1A, both genes are predictive of favorable OS, suggesting interconnected roles in melanoma immunosurveillance. Next, we extended our correlation analysis to all cancer types of the GDC-TCGA-PANCAN transcriptome dataset. Notably, *TIMP1* displayed a significant positive correlation with *CD8A* infiltration across most cancer types (Fig. 1B, left panel).

While correlations between immune genes and T cell infiltration markers can be a byproduct of mere co-expression during inflammation, if a given immune biomarker shows a strong correlation with inflammatory triggers, such as exclusive antigen-presentation biomarkers expressed in resident antigen-presenting cells (APCs), like DCs, it hints at a potential functional immunogenicity role of this biomarker. Thus, a substantial correlation of *TIMP1* with immunogenic triggers, such as *HLA* genes would support its functional link to increased *CD8A* levels in the TME; moreover, a weaker correlation could indicate co-expression over potential causation. Notably, in the GDC-TCGA-SKCM dataset, *TIMP1* displayed strong correlations with *HLA-DRA*, an antigen-presentation marker by DCs (MHC-II in mice), in addition to a cytolytic activity signature of CD8 + T cells [41], and *CD3E* T cell infiltration marker (Supplementary Fig. 1A).

We expanded this correlational study using a collection of *HLA* genes represented in a *TIMP1* supervised heatmap classification (Supplementary Fig. 1B), observing their strong association with *TIMP1*. Next, we performed Spearman’s rank test of *TIMP1* with these genes, notably highlighting significant ties with *HLA-DMA* and *TPBP* (Fig. 1B, right panel). Importantly, the *TPBP* gene encodes tapasin, which bridges the TAP complex and MHC-I molecules, optimizing peptide loading onto MHC-I for presentation to cytotoxic T cells [42].

Since it has been established that CD8 + T cell-derived IFN-γ can potentiate the expression of MHC-I (HLA-A) [43], the predominant expression of *HLA-A* in the tumor biopsy could solely be a consequence of IFN-γ signaling. To address this hypothesis, we conducted a focused analysis on a subset of patient TCGA-SKCM and PANCAN biopsies devoid of *IFNG* expression. Contrary to the assumption that *HLA-A* expression is predominantly a consequence of *IFNG* levels, our findings reveal a not only significant but increased correlation between *TIMP1* expression and the level of *HLA-A*. Specifically, we observed a Spearman’s correlation coefficient increase from r = 0.244 to r = 0.335 in the SKCM dataset, and r = 0.545, *p* < 0.0001 in the PANCAN dataset (Supplementary Fig. 1C), affirming the strength of this association independent of *IFNG* levels. These data suggest a functional regulatory axis between *TIMP1* and *HLA* molecules that may influence immune surveillance mechanisms within the tumor microenvironment.

To qualitatively evaluate the functional relationship between *TIMP1* and *HLA* genes, we undertook a Force Atlas 2 network analysis, which is an effective tool for converting correlative data into insights indicative of functional relationships [44]. The algorithm works by revealing clusters of closely positioned genes, hinting at shared functional biological processes. In our analysis, we integrated the gene for the Human Homeostatic Iron Regulator Protein (*HFE*), also known to affect peptide loading onto unstable MHC molecules [45], as a functional reference control. Despite neither *TIMP1* nor *HFE* being *HLA* protein family members, *TIMP1* showed a closer relation to *HLA* nodes compared to *HFE* (Fig. 1C). Due to *HFE’s* functional nature, the higher attraction of *TIMP1* to *HLA* nodes suggests a functional role for TIMP-1 in antigen presentation. Therefore, these findings suggest that the role of TIMP-1 goes beyond mere co-expression seen in bulk RNA datasets, suggesting a more functionally relevant correlation, possibly confined to immune segments of the tumor microenvironment.

### Spatial NGS analysis of lymph nodes from metastatic melanoma patients reveals TIMP-1 association with HLA and CTL Signatures and CD8 + T Cell differential levels

Next, we hypothesized that if *TIMP1* functionally contributes to antigen presentation, its correlation with *HLA* genes should be observed not only in the TME but also in lymphoid organs, specifically within immune infiltrates. To test this, we used TMA cores from skin and tumor-draining LN of metastatic melanoma patients. The process of obtaining human tissues, and evaluating H&E stainings for NanoString GeoMX access is depicted in Fig. 2A. We applied the spatial NanoString Geo MX profiling and NGS in tumor and iROIs, which were phenotyped by H&E and Immunofluorescence stainings (Fig. 2B, upper panel, and Supplementary Fig. 2A) as previously described [23]. Upon data normalization and conducting quality control analysis, we carried out a Principal Component Analysis (PCA) on the entire transcriptome, revealing two primary clusters that can be distinguished by high and low *TIMP1* expression correlated genes clusters (Supplementary Fig. 2B).

We then discerned that significant positive correlations between *TIMP1* and *CD8A* were confined to instances where *TIMP1* was expressed in the immune segment (highlighted in red), rather than with *TIMP1* expression in the tumor segment (depicted in green) (Fig. 2B, lower correlation panels). This finding implies that the presence of *TIMP1*-expressing cells within the immune compartment is linked to the development of more immunogenic tumor types. Further investigation into the differential expression of *TIMP1* across immune and tumor regions revealed that heightened *TIMP1* expression predominantly occurs in the immune segments of patients with overall high systemic *TIMP1* levels (Fig. 2C). This observation underscores that, although *TIMP1* RNA is detectable in both tumor and immune areas, its enhanced expression in the immune segments of highly immunogenic tumors may reflect functionalities divergent from those manifested in tumor cells.

Due to the significant relevance of *TIMP1* in the LNs, we further evaluated the correlation of *TIMP1* with *HLA* genes like *HLA-A*, *-B*, *-C*, *TPBP*, and *TAP1* (Fig. 2D, upper panel), with *TPBP* notably matching our previous findings in GDC-TCGA-SKCM dataset. These insights suggest a role for *TIMP1* not only associated with the expression of HLA-A molecules but with genes involved in the processing and expression control of HLA, such as *TBPA* and *TAP*, suggesting a functional link for TIMP-1 in lymphoid organs. Next, we assessed whether higher *TIMP1* expression in iROIs of LNs associated with improved antigen presentation and T-cell generation. This was done by examining a combination of *HLA* genes and a gene signature that indicates the level of CD8 + T cell cytolytic activity on the biopsy (*CTL* signature) as previously described [41]. Elevated *TIMP1* levels significantly correlated with upregulated signatures, suggesting more active antitumor T cell generation (Fig. 2D, lower panel) [41]. These findings indicate that *TIMP1* is not merely associated with *HLA-A* expression, but it encompasses genes integral to HLA processing and expression, like *TAPBP* and *TAP*, hinting at a potential functional significance of *TIMP1* expression within the TME and lymphoid tissues to prime antitumor effector T cells.

Next, we divided our internal cohort into groups with high and low levels of *CTL* signature. Using Force Atlas2, we analyzed the network dynamics, focusing on the interaction intensity and nodal proximity between *TIMP1* and *HLA* genes. *TIMP1* showed closer proximity to *HLA* nodes in LNs with high *CTL* levels (Fig. 2E). These findings suggest that the role of TIMP-1 goes beyond mere co-expression seen in bulk RNA datasets, suggesting a more functionally relevant correlation, possibly confined to immune segments of the tumor microenvironment. Therefore, we hypothesize a functional link for TIMP-1 influencing antigen presentation and the generation of cytolytic CD8 + T cells in LNs of metastatic melanoma patients, which may stem from its cellular and molecular functions on local immune cells.

### *TIMP1* cross-tissue correlation is associated with an immunogenic gene signature in the lymph nodes with implications for MHC-I peptide loading

**Fig. 3:**
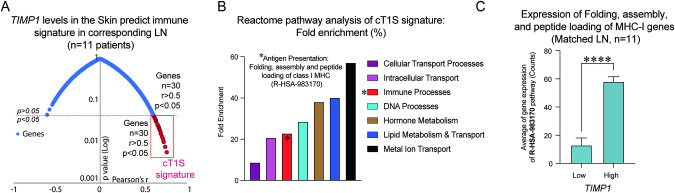
Cross-tissue correlation of *TIMP1* and gene enrichment analysis from cT1S signature metastatic melanoma. **A** Correlation-Significance Mapping Scatter Plot of the cT1S signature. RNA levels of *TIMP1* from iROIs in skin tumors were tested with the entire transcriptome from iROIs in the tumor-draining lymph nodes from 11 melanoma patients. The red box depicts the 30 genes with significant (at least *p* < 0.05) positive correlation from Pearson’s test within the transcriptome of tumor-draining lymph nodes. **B** Gene enrichment analysis of the cT1S signature using PANTHER classification system. The analysis aimed to characterize the functional implications of the cT1S signature, highlighting its distribution across different biological pathways. Full name of the detected immune pathway: Antigen Presentation: Folding, assembly, and peptide loading of MHC-I (R-HSA-983170). **C** Average expression from all genes belonging to R-HSA-983170 category according to *TIMP1* differential expression in matched tumor-draining lymph nodes. Cutoff for *TIMP1* determined by the median of expression counts within the cohort.

In prior studies on parasitic intracellular infection, myeloid DCs were found to express and secrete TIMP-1, enhancing their migratory and differentiation abilities [46]. Given the similarities between immune responses to intracellular infections and cancer immunosurveillance, particularly in the context of antigen presentation and Th1-type immune response [47, 48], we postulated that TIMP-1 might influence the activation and migration of tumor-resident APCs to tumor-draining LNs. Thus, levels of *TIMP1* in the TME should correlate with immune biomarkers in the lymphoid tissues due to the role of activated tumor-resident DCs in antigen presentation. To validate this, we compared the transcriptomes from iROIs of skin tumors and matched tumor-draining LNs in 11 metastatic melanoma patients. Of the 10,636 genes analyzed, only 30 genes from the LNs showed a significant correlation with *TIMP1* levels in the skin iROIs, forming the cross-tissue *TIMP1* correlated signature (cT1S) depicted in an inverted volcano plot (Fig. 3A, and Supplementary Table 2).

Subsequently, we carried out a gene enrichment analysis to interpret the potential immunological implications of the cT1S signature in the tumor-draining LNs using Reactome pathway analysis (Supplementary Table 3), as described in the materials and methods. We found that among seven subcategories influenced by cT1S, Metal Ion Transport had the highest enrichment scores, while DNA and Immune Processes showed intermediate scores (Fig. 3B and Supplementary Table 3). Notably, the sole representative category for Immune Processes was the *“Antigen Presentation: Folding, assembly, and peptide loading of class I MHC (R-HSA-983170)”* pathway (Fig. 3B and Supplementary Table 4). We further observed a robust upregulation of this pathway in LNs with high *TIMP1* expression (Fig. 3C). These findings expand the functional links for *TIMP1* expression in the TME, which is not only associated with the activation and migration of local DCs to LNs [49], as previously described, but it modulates the machinery responsible for processing antigens and load to MHC-I molecules in the context of cancer immunosurveillance. With this evidence, we undertook a functional study to confirm the role of TIMP-1 in DC activation.

### Myeloid DCs secrete TIMP-1

**Fig. 4:**
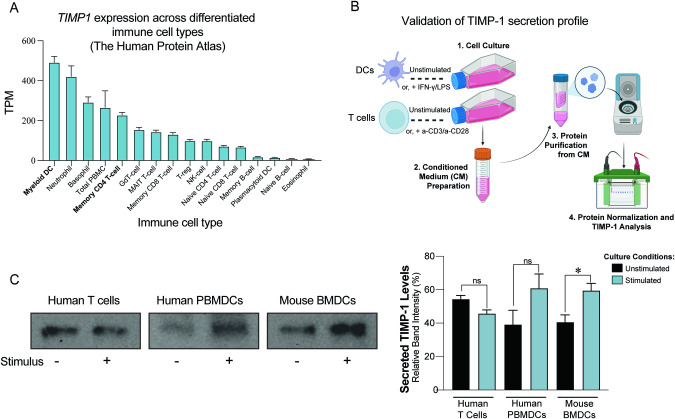
Characterization of TIMP-1 expression and secretion in different cell types. **A** Characterization of *TIMP1* RNA expression across differentiated immune cells. Data represents the normalized average of RNA-seq counts from the dataset of the Human Protein Atlas (HPA), Monaco [51], and Schmiedel [52] cohorts. **B** Schematic overview depicting the evaluation of soluble TIMP-1 levels in conditioned media derived from primary DCs (both mouse and human) and primary human T cells, comparing unstimulated and stimulated states for further purification and evaluation by Western blot. **C** Western blot analyses display TIMP-1 protein levels (left). Protein quantities were normalized before gel loading, with subsequent relative quantification of TIMP-1 conducted against the total protein loaded, as depicted in the bar charts (right). Three independent experiments are shown, mean ± SEM from combined biological replicates, two-tailed unpaired *t* test (*p < 0.005; ns: not significant).

Recent research shows that DCs infected with *Toxoplasma gondii* become activated and migrate due to autocrine TIMP-1 stimulation [49]. Therefore, we examined *TIMP1* expression in differentiated immune cells that compose the diverse immune landscape of the TME [50], such as DCs, macrophages, T cells, neutrophils, and NK cells. Using the single-cell transcriptome dataset from the Human Protein Atlas (HPA) portal, which merges data from *HPA, Monaco, and Schmiedel* cohorts [51, 52], we focused only on differentiated immune cells, thus excluding monocytes. We observed that myeloid DCs have the highest *TIMP1* mRNA levels and memory CD4 + T cells express moderate levels of *TIMP1* (Fig. 4A).

We then studied TIMP-1 secretion in the protein fraction from the conditioned media of primary cultures of DCs and T cells purified from PBMCs of healthy donors using immunoblotting (Fig. 4B). We observed that secreted TIMP-1 is indeed present in the condition media of steady-state human DCs and T cells (Fig. 4C). These findings support existing knowledge of autocrine TIMP-1 produced by immune cells [49, 53]. To investigate the dynamics of soluble TIMP-1 levels under different activation states, we stimulated human DCs with IFN-γ and LPS, and T cells with anti-CD3 and anti-CD28 antibodies. Our findings show a qualitative but not significant increase of secreted TIMP-1 levels only in stimulated human DCs. This preliminary observation led to testing whether specific subpopulations of DCs pertinent to tumor antigen presentation could manifest better TIMP-1 secretion in response to stimulus. To test this, primary mouse BMDCs were obtained using the FLT3L differentiation method [27], which enhances their proficiency in tumor antigen presentation for anti-PD1 responses [28], and then stimulated primary cultures using LPS. Following the pattern observed in peripheral human DCs, we detected a notable and significant increase in soluble TIMP-1 from BMDCs upon stimulation (Fig. 4C), suggesting TIMP-1 secretion upon stimulus.

### Soluble TIMP-1 elicits MHC-I expression in myeloid DCs

**Fig. 5:**
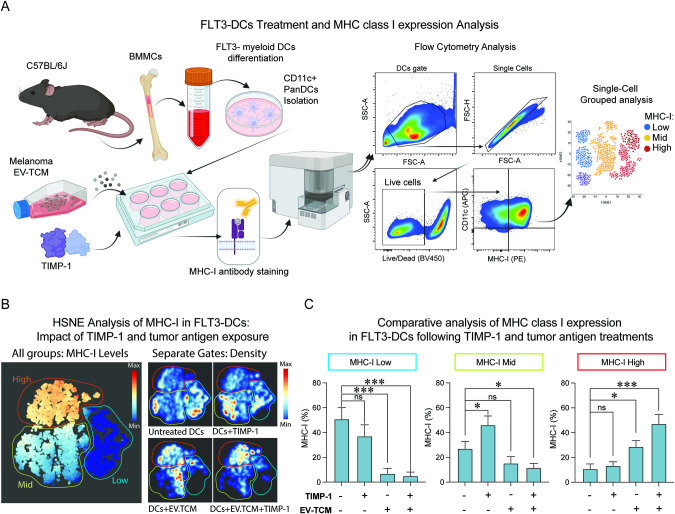
Influence of TIMP-1 on MHC-I expression levels in myeloid DCs. **A** Schematic representation of the functional study to evaluate TIMP-1 impact on MHC-I levels in myeloid DCs. Flow cytometry analysis of surface MHC-I expression on primary myeloid DCs under different conditions: 1:4 EV-TCM from B16F10 cells, recombinant TIMP-1, or a combination of both. **B** left Hierarchical Stochastic Neighbor Embedding (HSNE) plots for unsupervised clustering of the three distinct MHC-I expression patterns: high (red), intermediate (green), and low (blue) expression levels. **B** right Single-cell density analysis within each MHC-I cluster for each experimental group. Normalized frequencies of live myeloid DCs across low (**C**, left), mid (**C**, middle), and high (**C**, right) MHC-I expression levels. Three independent experiments are shown, mean ± SEM from combined biological replicates, two-tailed unpaired *t* test (***p < 0.001; *p < 0.05; ns: not significant).

To investigate the role of soluble TIMP-1 in myeloid DC activation, we studied its influence on MHC expression as indicative of its potential impact on antigen presentation. We treated FLT3L-differentiated myeloid BMDCs with recombinant mouse TIMP-1 validated in previous studies [29–31, 49]. Moreover, TIMP-1 was also added into the BMDC culture in combination with a vesicle-enriched tumor-conditioned media (EV-TCM) from 72-h cultured B16F10 melanoma cells, known for effectively enriching melanoma antigens for MHC-I-mediated CD8 + T cell priming [54]. MHC-I expression on the surface of DCs was then evaluated by flow cytometry (Fig. 5A). Representative gating strategy and scatter-plots of the expression levels of MHC-I are shown in (Supplementary Fig. 3A).

To provide more robustness in our quantification, we concatenated our data from three independent experiments, as previously described [36], and used Cytosplore to perform HSNE clustering. This analysis allowed an in-depth portrayal of MHC-I expression through unsupervised density clustering of single cells, discerning three MHC-I expression groups: high, intermediate, and low (Fig. 5B, left panel). Our analysis showed that most untreated DCs clustered within the low MHC-I expression. Intriguingly, TIMP-1 shifted expression toward intermediate MHC-I levels, and in combination with EV-TCM, it intensified MHC-I expression (Fig. 5B, right panel). Single-cell quantification highlighted that both EV-TCM and its combination with TIMP-1 markedly reduced the frequency of DC populations expressing low MHC-I (Fig. 5C, left panel). Conversely, TIMP-1, especially in combination with EV-TCM, amplified intermediate (Fig. 5C, middle panel) and high MHC-I expressions (Fig. 5C, right panel). These findings underscore the potential of TIMP-1 to act as a cytokine that enhances MHC-I expression on FLT3L-stimulated DCs, suggesting its beneficial role in antigen presentation.

To evaluate whether the obtained results were specific to TIMP-1 and not due to potential effects emerging from the low levels of endotoxin found in the recombinant protein used, we performed the same experiment using a non-functional heterologous control protein (recombinant human-TIMP-1 control) at a concentration that reaches the same endotoxin levels found in the species-specific TIMP-1. In such conditions, we observed that the endotoxin levels are not sufficient to induce an increase of MHC-I, reinforcing that the results obtained are specific to TIMP-1. Moreover, TIMP-1 did not induce the upregulation of the maturation marker CD86 (Supplementary Fig. 3), suggesting that the observed effects are not due to generalized DC maturation or activation, but potentially modulating the antigen-processing machinery of DCs (Supplementary Fig. 3A, left panels). Notably, recent studies have highlighted the essential role of the cDC1 subset in mediating the effectiveness of immune checkpoint therapies [55]. In this context, our study also examined the response of MHC-I levels to TIMP-1 stimulation in this particular DC subtype. Our results demonstrate that TIMP-1 exerts stimulatory effects on these cells (Supplementary Fig. 3A, right panels), a factor that could be pivotal in determining the outcomes of immunotherapeutic approaches. Furthermore, relative MFI analysis revealed that TIMP-1 alone indeed leads to increased MHC-I expression compared to untreated control in both total CD11c+ BMDCs, and the cDC1 subtype (Supplementary Fig. 3B).

Understanding the well-established role of MHC-I in CD8 + T cell activation [56], we sought to confirm the functional significance of increased MHC-I expression in DCs in the context of CD8 + T cell activation. Our pilot approach involved TIMP-1-treated FLT3L-BMDCs pulsed with ovalbumin (OVA) to facilitate cross-presentation, a process that delivers extracellular antigens to the MHC-I pathway for priming of CD8 + T cells [57]. This setup was then utilized to evaluate CD8 + T cell activation by quantifying both T cell proliferation by flow cytometry and IL-2 secretion in the culture supernatants (Supplementary Fig. 4A, B). Importantly, this preliminary approach allows us to observe the small fraction of clonally expanded T cells to soluble antigens cross-presented by DCs, as not all CD8 + T cell clones will be primed by DCs, as previously described [58]. Results indicated that TIMP-1-activated DC corresponded with a small increase in CD8 + T cell proliferation (from 4.9% to 5.6%) in the G2 proliferation gate, with no changes observed in co-cultures not previously pulsed with OVA (Supplementary Fig. 4C). Moreover, co-cultures including DCs previously treated with TIMP-1 and OVA showed a qualitative, although non-significant increase of IL-2 levels, essential for T cell proliferation (Supplementary Fig. 4D), laying the groundwork for future detailed studies on the systematic impact of TIMP-1 on cross-presentation and CD8 + T cell generation.

### TIMP-1 impacts the immunoproteasome/TAP complex

**Fig. 6:**
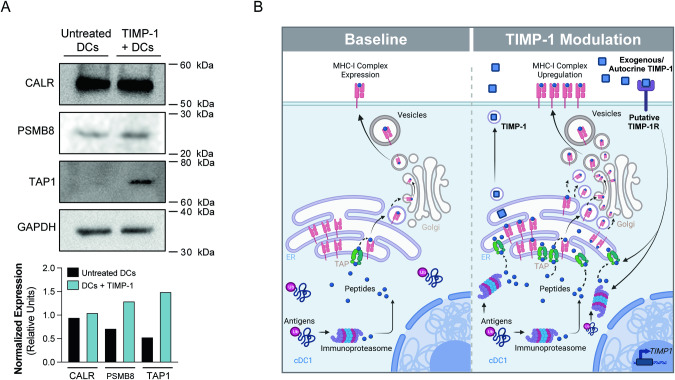
TIMP-1 impacts the immunoproteasome/TAP complex. **A** Western blot analyses display of calreticulin (CALR), PSMB8, and TAP-1 in whole cell lysates from BMDCs left untreated or treated with TIMP-1 (upper panel). Relative protein quantification was conducted against the total levels of GAPDH loading control, as depicted in the bar charts (lower panel). **B** Schematic representation of TIMP-1 modulatory effects on the immunoproteasome/TAP complex with implications for improved expression of MHC-I.

Building on the discovery that TIMP-1 augments MHC-I expression in BMDCs and aligning with our NanoString spatial analysis suggesting a role in antigen processing and loading to MHC-I molecules (Fig. 3), we further investigated how TIMP-1 stimulation alters the expression of critical proteins in the MHC-I peptide loading pathway. This included calreticulin with its role in peptide folding and loading onto MHC-I, PSMB8 with its importance in antigenic peptide generaation as part of the immunoproteasome, and TAP-1 which holds critical function in peptide translocation to the ER, alongside TAP-2. Following TIMP-1 stimulation, we observed that calreticulin levels remained unchanged, while slight increases in PSMB8 and more notable rises in TAP-1 were detected (Fig. 6A, upper panel). Expression levels relative to GAPDH loading control are represented in bar plots (Fig. 6A, lower panel). These results suggest that soluble TIMP-1 may indeed have an impact on processes that modulate the levels of the immunoproteasome/TAP complex, thereby boosting antigen processing and MHC-I expression for optimal peptide assembly and presentation on the cell surface (Fig. 6B).

## Discussion

TIMP-1 has emerged as an inflammatory cytokine in both innate and adaptive immunity [59]. Our study offers evidence for a novel immune function for TIMP-1, backed by its link with immunogenic characteristics of metastatic melanoma and favorable prognosis. *CD8A* expression in the TME is a positive indicator for cancer immunosurveillance, signifying the presence of cytotoxic T lymphocytes or CD8 + T cells, which are central to anti-cancer immunity [60]. Identifying molecules which serve as biomarkers correlating with *CD8A* and aid CD8 + T cell generation is crucial [6]. Herein we show that at the RNA level, *TIMP1* expression does correlate with *CD8A* expression in the TME of several cancer types of the PANCAN study, extending its pro-inflammatory roles in cancer.

The pronounced affinity of *TIMP1* to *HLA* genes compared to the *HFE* gene in our Force Atlas 2 analysis [44] underpins a functional link for potential *HLA*-related immunogenic roles at the level of APCs, as correlations restricted to immune segments within the biopsy and the lymphoid tissues. This connection emphasizes the importance of examining the intricate functional interplay of TIMP-1 in HLA expression, given the critical role of early DCs activation in cancer immunity and ICT responses [61]. Indeed, molecular dynamics in the TME have downstream consequences in the tumor-draining lymphoid tissues, where critical T cell priming events typically occur [62].

Our cross-tissue study highlighting *TIMP1* differential expression in the TME and its association with a gene signature indicative of peptide loading onto MHC-I molecules in tumor-draining LNs underscores a pivotal functional link for TIMP-1 in tumor immunogenicity.

Our findings advance the understanding of TIMP-1 secretion, demonstrating that myeloid DCs, in addition to T cells, are significant producers of TIMP-1. In addition, TIMP-1 secretion might indeed occur in response to stimulatory conditions, particularly by subpopulations of immunogenic DCs that are important for antigen presentation and immunotherapy outcomes. While the increase of TIMP-1 secretion by stimulated human DCs wasn’t statistically significant, it suggests a potential avenue for future studies, especially in conditions mimicking the tumor’s cytokine milieu or involving co-stimulatory immune signals. These findings, although preliminary, align with prior studies linking TIMP-1 to myeloid DC activation in the context of parasitic infection [49], and T cells as an additional source of soluble TIMP-1, from which secretion might occur in response to different dynamics within the TMA. Future studies will aim to precisely identify the cellular and molecular entities that significantly influence the autocrine secretion of TIMP-1 by human DCs and T cells, particularly in the context of the TME.

This supports the emerging view that TIMP-1 plays an autocrine role in enhancing DC immunogenicity. For the first time, we provide evidence that the functions of soluble TIMP-1 extend beyond its established role in DC migration during parasitic infection [49], to include the modulation of DC-mediated presentation of tumor antigens to T cells. This autocrine activity of TIMP-1 positions it alongside other key immunomodulatory cytokines such as IFN-γ and TNF-α [63], known for their efficacy in tumor immunity. Furthermore, the secretion of TIMP-1 by T cells underlines a potential supportive role for TIMP-1 in coordinating Th1 immune responses, since Th1 lymphocytes are known to impact the expression of MHC-I molecules on DCs through secretion of pro-inflammatory cytokines, such as IFN-γ and TNF-α, enhancing their cross-presentation capabilities [64, 65].

Our results indicate that soluble TIMP-1 augments MHC-I expression in myeloid DCs when exposed to tumor antigens, underscoring its potential role in modulating tumor immunity. While existing studies on TIMP-1’s effects on myeloid DCs are sparse, one notable research demonstrated its involvement in enhancing DC migration in response to *Toxoplasma gondii* infection [49]. Given the critical role of DC migration in tumor antigen processing and cross-presentation within lymphoid tissues [66], and considering the shared Th1 immune mechanisms employed against both cancer and intracellular infections, TIMP-1 functions in DC activation during infection may also be significant in cancer immunology.

Our study demonstrates that TIMP-1 enhances MHC-I expression in myeloid DCs, with an even more marked increase observed when these cells are co-incubated with TIMP-1 and exogenous antigens, suggesting an improved potential for antigen processing and cross-presentation. It is important to understand that effective antigen presentation encompasses more than just antigen processing and folding to MHC-I molecules for further surface expression; it also requires successful T-cell priming and activation. The initial findings suggest that TIMP-1 plays a role in boosting MHC-I expression, especially in the presence of exogenous antigens, highlighting it as a key precursor for further effective antigen presentation. Ongoing research in this area is expected to further validate successful antigen presentation via T-cell activation.

The exact mechanism through which TIMP-1 influences MHC-I is not fully elucidated. While it is evident that TIMP-1 impacts the immunoproteasome/TAP Complex [67, 68], the precise mechanism of this modulation is not yet understood. Nevertheless, our findings suggest that the impact of TIMP-1 on MHC-I expression could be due to the relocalization of MHC-I from the ER upon peptide loading by TAP proteins [67], however, this does not exclude the possibility for an increase in MHC-I expression, since optimized processed antigens implicate a need for increased MHC-I levels in the endoplasmic reticulum.

At a molecular level, TIMP-1 interacts with several receptors through its dual-domain structure [69]. Its N-terminal domain inhibits MMPs [70] and binds with CD82 [12], while both domains are associated with ADAM-10 [71]. There is evidence of TIMP-1 binding to LDL receptor-related protein 1 (LRP1) [13], but the details are yet to be delineated. A spotlight has been cast on the CD63 and CD74 receptors. In 2006, CD63 was identified as the first receptor interacting with soluble TIMP-1 [12]. Subsequent research showed the effects of TIMP-1/CD63 interaction on immune functions [72–74], including its influence on DCs, macrophages, and NK cells [49, 75, 76]. In DCs, TIMP-1 has been described to target CD63 and influence DCs migration through activation of ITGB1-FAK signaling [49]. However, this interaction might also play a pivotal role in MHC-I expression influenced by TIMP-1, especially in antigen presentation contexts.

In APCs such as B cells and DCs, the arrangement of MHC-I and MHC-II on the cell membrane is regulated by exosomes [77]. This arrangement is guided by tetraspanin enrichment microdomains (TEMs), which include CD63 and CD81 [78–80]. Importantly, studies have demonstrated that a reduction in CD63 levels within APCs augments the production of exosomes, leading to an improved arrangement of MHC-II on the cell membrane, thereby enhancing T-cell activation [81]. While both MHC-I and MHC-II arrangement on the cell membrane is coordinated by TEMs, it is yet to be clarified whether a reduction in CD63 might likewise enhance MHC-I cell surface localization. One hypothesis is that TIMP-1, by targeting [12] and potentially neutralizing CD63-dependent endosomal regulation, could enhance MHC-I expression. This may occur through amplified cross-presentation dependent on endosomes or increased exosome production in myeloid DCs exposed to higher TIMP-1 levels. However, this hypothesis, linking reduced CD63 activity to augmented MHC-I expression via TIMP-1 interaction, needs further research for comprehensive elucidation.

CD74 in DCs has also emerged as a promising molecular target for TIMP-1. In B cells, recent studies have shown that TIMP-1 targets CD74 and activates ZAP-70 signaling, increasing B cells activation functions [14]. While CD74 typically acts as a chaperone for MHC molecules under normal conditions [82, 83], it gets targeted by MIF in metastatic melanoma [84], altering its immunogenic role and reducing the DCs’ ability to expand antitumor CD8 + T cells [17]. We discovered that blocking MIF-CD74 interaction with an antitumor peptide (C36L1) can restore the immunogenic function of myeloid DCs [17]. Notably, C36L1 can obstruct the interaction between CD74 and TIMP-1 [14], suggesting TIMP-1 could be a potential CD74 ligand with implications on DC immunogenicity, with similar implications for DC immunogenicity as observed with C36L1 peptide. Given the link between TIMP-1 and enhanced immunogenicity in melanoma, its interaction with CD74 might either bolster its MHC-I/II chaperoning role or compete with MIF, reducing MIF-induced suppressive effects through CD74. This hypothesis calls for deeper therapeutic exploration, especially considering that TIMP-1 influences MHC-I levels on DCs.

Therapeutically, leveraging MHC-I expression potentiates ICT outcomes [85]. On this front, the power of mRNA vaccines to introduce immunogenic genes in the TME offers novel combinatorial avenues for immune modulation. Just as mRNAs encoding CD70, CD40 ligand, and TLR4 [6], have been investigated for this purpose, TIMP-1 could be similarly delivered targeting resident DCs. By using mRNA vaccines to induce TIMP-1 expression in the TME, we might enhance DC activation, and transition cold tumors to hot ones by optimizing T cell generation in the lymphoid organs to improve ICT outcomes.

## Conclusion

As cancer immunology progresses, understanding diverse immune cells and their regulators is essential. Utilizing a broad approach to study factors like environmental impacts and epigenetics is key for targeting ICT-resistant cold tumors. TIMP-1 is crucial in driving both adaptive immunity through processes that modulate the immunoproteasome/TAP complex, increasing MHC-I expression in cDC1 subset (Fig. 6B), which are fundamentally important for ICT outcomes [55]. These findings thus identify TIMP1 as a potential new target for reshaping cancer immunotherapy. Exploring its roles may lead to enhanced strategies benefiting not just melanoma, but other cancers where TIMP-1 correlates with improved survival. The central aim is optimizing tumor immunogenicity for better ICT outcomes in cold tumors. The strengths of this study include solid clinical evidence on the association of TIMP-1 with HLA expression and improved CD8 T cell levels, both in the tumor and in LN of melanoma patients. This association is further dissected at the spatial level, enabling functional links with antigen processing and loading machinery to MHC-I, further validated with key functional studies.

One potential limitation of this study may be the unavailability of extensive in vivo studies that delve into the intricate mechanistic aspects of TIMP-1’s influence on tumor immunity. Additionally, more robust studies are needed to comprehensively understand the functional impact of TIMP-1-induced cross-presentation by DCs for consequent priming and activation of CD8 + T-cell responses. This can be done by testing different TIMP-1 stimulatory conditions, using antigen-specific CD8 + T cell-based cross-presentation assays and evaluating a broader spectrum of parameters—such as activation markers on both DCs and CD8 + T-cells, and cytokine profiling.

This study lays the initial groundwork for further exploration into how TIMP-1 modulates tumor immunogenicity, particularly in light of MHC-I-dependent antigen presentation. Our findings offer a clear direction for upcoming research that seeks to decode the complex molecular and cellular interactions driven by TIMP-1. Future investigations are vital for a deeper understanding of these mechanisms and will inform the development of novel combinatorial therapeutic approaches to enhance the immunogenicity of cold tumors, ultimately refining the effectiveness of current immunotherapy treatments.

### Supplementary information

Supplementary Material Captions

Supplementary Figure 1

Supplementary Figure 2

Supplementary Figure 3

Supplementary Figure 4

Supplementary Table 1

Supplementary Table 2

Supplementary Table 3

Supplementary Table 4

## Data Availability

PANCAN and SKCM transcriptomic RNA-seq data are available at the GDC portal (https://portal.gdc.cancer.gov), and normalized cases can be downloaded from the UCSC Xena platform (https://xenabrowser.net/). *TIMP1* RNA-seq from different immune cells is available at the Human Protein Atlas (proteinatlas.org) under the access code ENSG00000102265. NanoString GeoMX data is deposited in Figshare (10.6084/m9.figshare.24962505z).
