# Supplementary material for: TIMP-1 is an activator of MHC-I expression in myeloid dendritic cells with implications for tumor immunogenicity
Source: Genes Immun. 2024 May 22;25(3):188–200. doi: 10.1038/s41435-024-00274-7 (PMC11178497; doi:10.1038/s41435-024-00274-7)
Supplement: Supplementary file 1 — Supplementary Material Captions [file 41435_2024_274_MOESM1_ESM.docx]

**TIMP-1 is an activator of MHC-I expression in myeloid dendritic cells with implications for tumor immunogenicity.**

**Supplementary Figure Captions**

**Supplementary Figure 1. (A)** Spearman’s rank correlation of *TIMP1* expression with gene signatures predictive of cytolytic activity (CTL), *CD3E,* and *HLA-DRA* levels. (**B)** Heatmap of normalized RNAseq - HTSeq - FPKM-UQ gene expression scores, providing a qualitative view of the *TIMP1* and *HLA*-family gene interaction. **(C)** Spearman’s rank correlation of *TIMP1* and *HLA-A* in tumors with no/low *IFNG* expression levels in the (left) GDC-TCGA-SKCM cohort (n=57) (right) and GDC-TCGA-PANCAN (n=1858) cohort.

**Supplementary Figure 2. (A)** LN biopsy of melanoma metastasis patients (n=37) from the Auria cohort **(**left) stained with H&E, showing a representative intersection of immune and tumor cellular architectures (right) and matched visualization of the melanoma and immune regions of interest (iROIs) in the PMEL17+CD45- cells (green) and PMEL17-CD45+ (red), respectively. **(B)** Principal Component Analysis (PCA) plot of the human transcriptome of Auria LN cohort revealing two distinct clusters: *TIMP1* low (green) and *TIMP1* high (red), showcasing differential *TIMP1* expression within the dataset.

**Supplementary Figure 3. (A)** Gating strategy followed for analyzing MHC-I+ and CD86+ cells within total CD11c+ and cDC1 subset. Representative gates indicating the frequencies (%) of MHC-I+ and CD86+ within total CD11c+ and cDC1s. **(B)** MHC-I relative MFI calculated within total CD11c+ for untreated DCs, TIMP-1-treated DCs, and human TIMP-1-treated DCs (left), and within cDC1s for untreated DCs and TIMP-1-treated DCs (right). Four independent experiments are shown, mean ± SEM from combined biological replicates, two-tailed unpaired *t*-test (***p < 0.001; ns: not significant).

**Supplementary Figure 4**: CD8+ T cell activation and proliferation response to TIMP-1 stimulated DCs. **(A)** Workflow of *ex vivo* DC/CD8+ T-cell cross-presentation assay. **(B)** Flow cytometry gate strategy to quantify CMFDA-labeled CD8+ T cell proliferation. **(C)** Comparative analysis of T cell proliferation at early (G1) and late (G2) generation stages following ovalbumin (OVA) cross-presentation. Three independent experiments are shown, mean ± SEM from combined biological replicates, a two-tailed unpaired *t*-test. (*p < 0.05; ns: not significant). **(D)** Qualitative analysis of IL-2 protein secretion levels in culture supernatants, employing the TaqMan mouse IL-2 Immunoassay. IL-2 abundance is indicated by inverse Cq values (left) and quantified as standard IL-2 protein concentrations after normalization to total culture supernatant protein. Data combines three biological repetitions with six technical replicates, mean ± SEM from combined biological and technical replicates, two-tailed unpaired *t*-test.

**Supplementary Table Captions**

**Supplementary Table 1**: Gene expression of GDC-TCGA-SKCM melanoma patients. Scores are presented in log2(fpkm-uq+1) from gene expression RNAseq - HTSeq - FPKM-UQ data.

**Supplementary Table** **2:** Spearman's correlation coefficient and p values for *TIMP1* expression in skin melanoma tumors relative to the transcriptome of corresponding lymph nodes in patients with metastatic melanoma.

**Supplementary Table 3:** Top 20 reactome human pathways derived from T1S signature.

**Supplementary Table 4:** Gene list from folding, assembly, and peptide loading of class I MHC (R-HSA-983170) reactome pathway.
