# Supplementary figures and images for: TIMP-1 is an activator of MHC-I expression in myeloid dendritic cells with implications for tumor immunogenicity
Source: Genes Immun. 2024 May 22;25(3):188–200. doi: 10.1038/s41435-024-00274-7 (PMC11178497; doi:10.1038/s41435-024-00274-7)

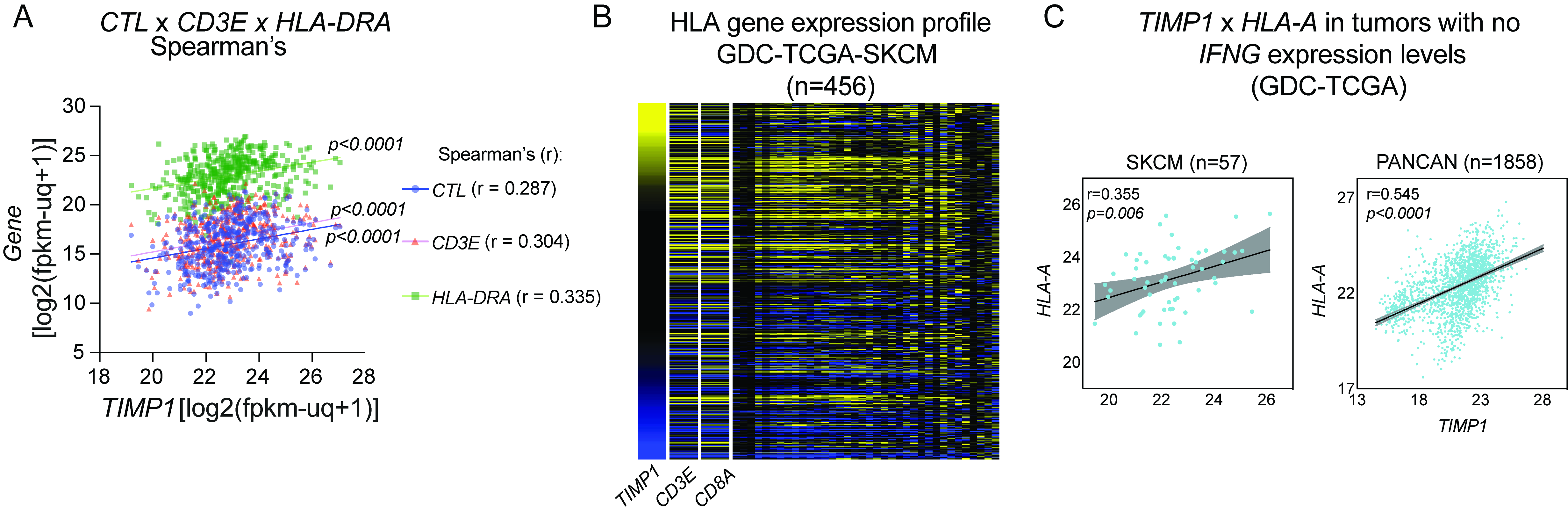

Supplement: Supplementary file 2 — Supplementary Figure 1 [file 41435_2024_274_MOESM2_ESM.tif]

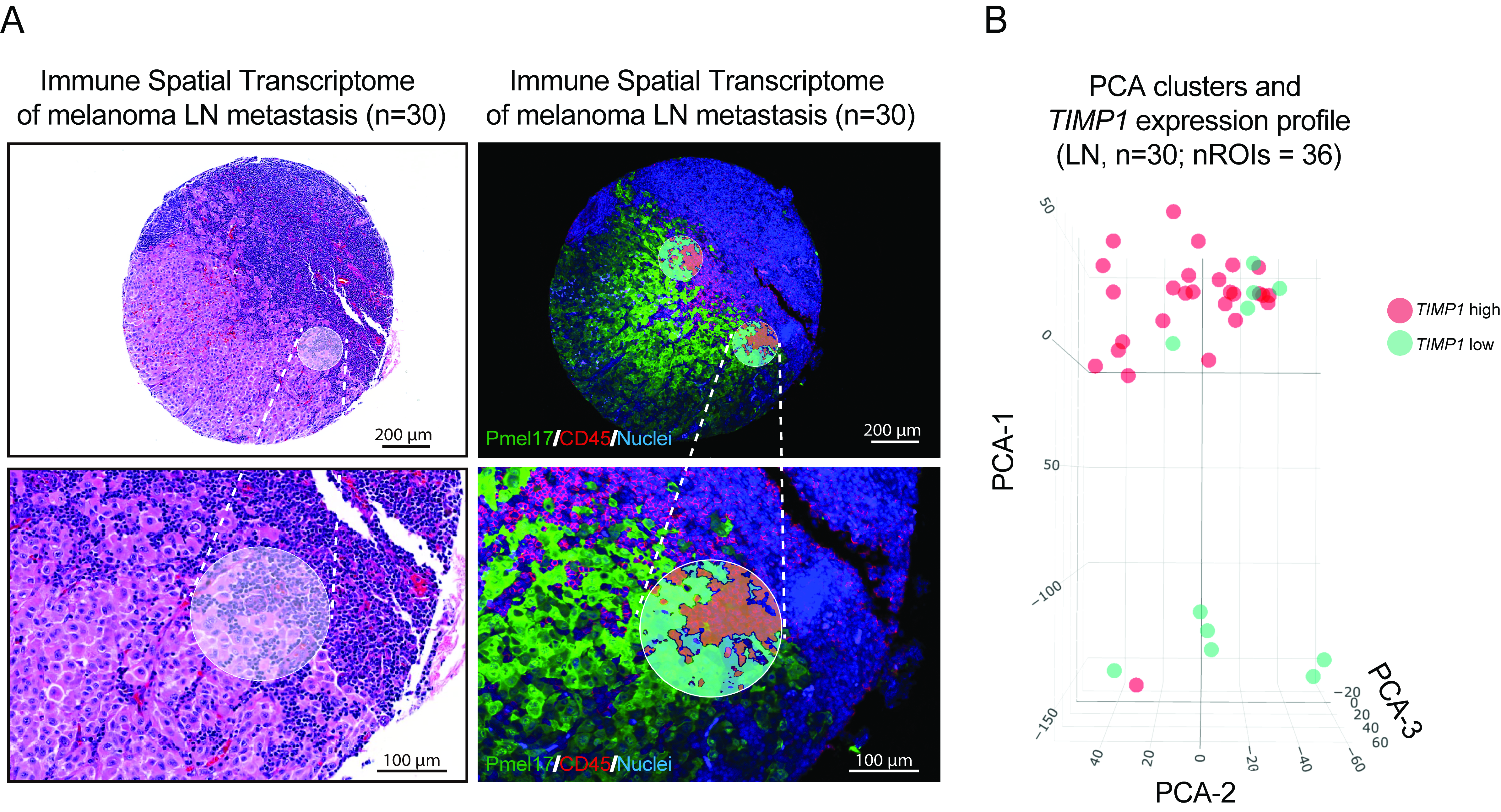

Supplement: Supplementary file 3 — Supplementary Figure 2 [file 41435_2024_274_MOESM3_ESM.tif]

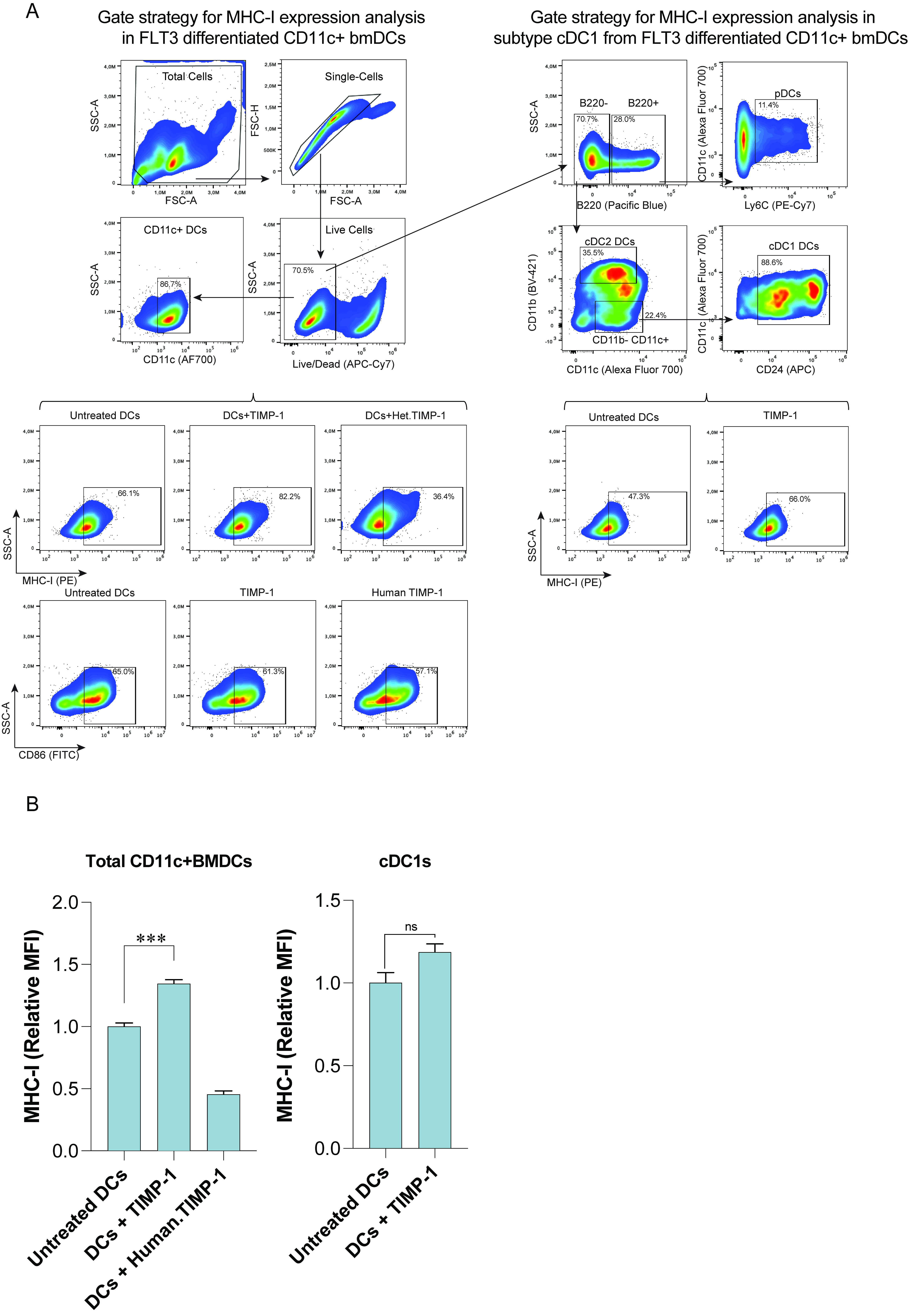

Supplement: Supplementary file 4 — Supplementary Figure 3 [file 41435_2024_274_MOESM4_ESM.tif]

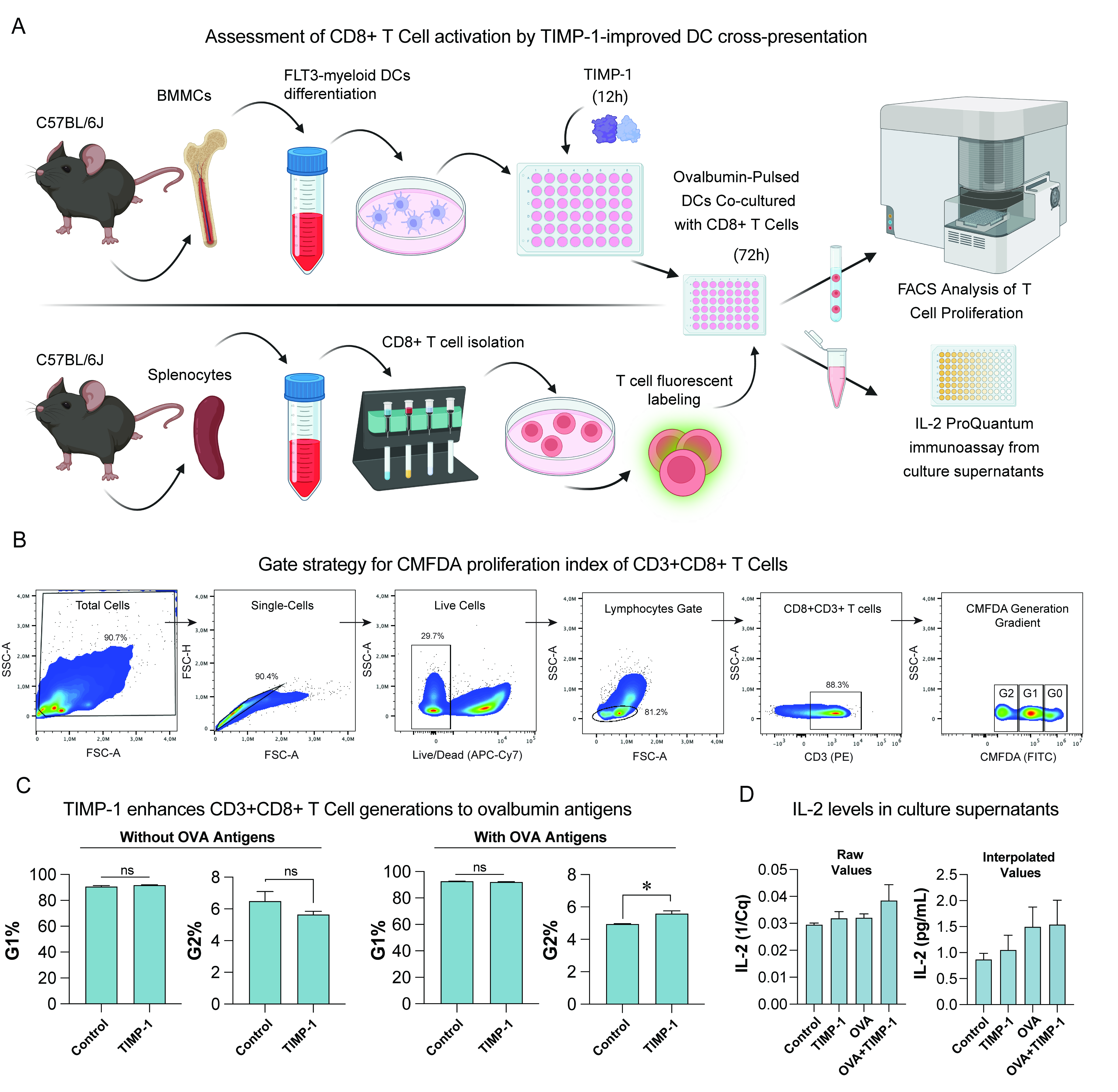

Supplement: Supplementary file 5 — Supplementary Figure 4 [file 41435_2024_274_MOESM5_ESM.tif]
